# Novel targeted agents for gastric cancer

**DOI:** 10.1186/1756-8722-5-31

**Published:** 2012-06-18

**Authors:** Lian Liu, Ning Wu, Jin Li

**Affiliations:** 1 Department of Medical Oncology, Fudan University Shanghai Cancer Center, Shanghai, 200032, China; 2 Department of Oncology, Shanghai Medical College, Shanghai, 200032, China; 3 Department of Medical Oncology Xuhui District central Hospital, Shanghai, 200031, China

**Keywords:** Gastric cancer, Targeted therapy, Monoclonal antibody, Tyrosine kinase inhibitor

## Abstract

Contemporary advancements have had little impact on the treatment of gastric cancer (GC), the world’s second highest cause of cancer death. Agents targeting human epidermal growth factor receptor mediated pathways have been a common topic of contemporary cancer research, including monoclonal antibodies (mAbs) and receptor tyrosine kinase inhibitors (TKIs). Trastuzumab is the first target agent evidencing improvements in overall survival in HER2-positive (human epidermal growth factor receptor 2) gastric cancer patients. Agents targeting vascular epithelial growth factor (VEGF), mammalian target of rapamycin (mTOR), and other biological pathways are also undergoing clinical trials, with some marginally positive results. Effective targeted therapy requires patient selection based on predictive molecular biomarkers. Most phase III clinical trials are carried out without patient selection; therefore, it is hard to achieve personalized treatment and to monitor patient outcome individually. The trend for future clinical trials requires patient selection methods based on current understanding of GC biology with the application of biomarkers.

## Introduction

GC remains a major cancer burden across the globe. In 2008, approximately 989,000 new cases (7.8 % of global cancer totals), and 738,000 deaths (9.7 % of global cancer totals) occurred, making it the fourth most common malignancy and the second leading cause of cancer death worldwide[1]. Geographically, Asian and South American countries have a higher incidence rate of GC than the United States and Western Europe. Though the absolute incidence of GC has declined globally in the second half of the 20th century, the relative incidence of proximal GC has increased notably [2].

Traditional treatments, such as curative surgery, radiotherapy, and perioperative chemotherapy, may improve the survival rate of operable GC patients. However, most patients are either diagnosed at an advanced stage or are subject to relapse after prior curative surgery. For these advanced patients, 5-FU (5-fluorouracil) cisplatin, or their analogs remain standard treatment regimens, with or without an anthracycline [3].

In the past decade, targeted therapies have significantly impacted the treatment strategy of many common malignancies, including breast, colorectal, and lung cancers. Unfortunately, research shows fewer encouraging targeted treatments for GC than for other cancers. Recently, Trastuzumab has been approved as standard care for HER2-positive GC patients, according to the results of clinical trials using ToGA (Trastuzumab for Gastric Cancer) [4]. Many other molecular targeted agents are also currently undergoing clinical trials, including VEGF pathway targeting agents, other HER family targeting agents, mTOR pathway inhibitors [5], and histone deacetylase (HDAC) inhibitors [6]. This review discusses recent investigations of targeted agents for the treatment of advanced GC.

### The Pathway of Targeted Therapy

Based on clinical outcomes from other malignancies, many new treatment choices using targeted agents have been studied in GC. Most targeted therapies focus on the VEGF and epidermal growth factor receptor (EGFR) related indications in advanced GC. Compounds against novel targets, such as mTOR, c-Met (hepatocyte growth factor receptor), and HDAC are also under investigation. Table  1 lists current ongoing phase III trials of targeted agents designed for the treatment of advanced GC.

**Table 1:** Ongoing phase III trials of targeting agents for the treatment of advanced gastric cancer

**ClinicalTrials.gov Identifier**	**Setting**	**Location**	**Masking**	**Estimated enrollment**	**Primary endpoint**	**Arm**
NCT00824785	1^st^ line	UK	Open-label	730	OS	EOX, EOX + panitumumab
NCT01248403	2^nd^ line	Germany	Double blind	500	OS	Paclitaxel, Paclitaxel + everolimus
NCT01170663	2^nd^ line	Global	Double blinded	633	OS	Placebo + paclitaxel, Ramucirumab + paclitaxel
NCT00450203	1^st^ line	UK	Open Label	1100	OS	ECX, ECX + Bevacizumab
NCT00917384	2^nd^ line	Global	Double blinded	315	OS	Placebo + BSC, Ramucirumab + BSC
NCT01512745	3^rd^ line	China	Double blinded	500	PFS	Placebo, Apatinib

OS, overall survival; PFS, progression-free survival; EOX, epirubicin, oxaliplatin and capecitabine; ECX, epirubicin, cisplatin and capecitabine.

### Anti-VEGF/VEGFR Agents

Angiogenesis, the growth of new blood vessels, is an important aspect of tumorigenesis that not only provides tumor cells with nutrients and oxygen, but additionally serves as a pathway for tumor cells to enter the circulatory system, where subsequent metastasis may occur [7]. Tumor angiogenesis is primarily modulated by VEGF A and the receptors of VEGF (VEGFR) [8]. In GC, VEGF expression is related to tumor aggressiveness and is ultimately an indicator for poor prognosis [9-15]. Anti-VEGF agents have recently been developed, including mAbs and TKIs, for the reasons mentioned above.

### Bevacizumab

Bevacizumab is a VEGF A blocking mAb currently under investigation for the treatment of GC. Several phase II trials combining bevacizumab with different chemotherapeutic compounds were conducted on treatment-naive patients with locally advanced or metastatic GC, or with gastroesophageal junction cancer (GEJC), demonstrating results which were initially promising [16-18]. On the basis of results from these phase II studies, a phase III randomized, double-blind, contrast study (AVAGAST) was conducted internationally [19]. This study included 774 patients with previously untreated and locally advanced or metastatic GC or GEJC. Patients were treated with capecitabine and cisplatin in combination with either bevacizumab or a placebo. The median rate of overall survival (OS) was 10.1 months for the placebo group and 12.1 months for the bevacizumab group (HR = 0.87; P = 0.1002), failing to meet the primary endpoint; however, significant improvement in progression-free survival (PFS) and overall response rate (ORR) was noted in the bevacizumab group. In subgroup analysis, OS for the pan-American subgroup was 6.8 months for placebo vs. 11.5 months for bevacizumab (HR = 0.63). For European and Asian-Pacific subgroups, OS was 8.6 vs. 11.1 months (HR = 0.85), and 12.1 vs. 13.9 months (HR = 0.97), respectively, with all results favoring bevacizumab. These results indicate that Pan-American patients obtained the greatest benefit from the additional bevacizumab treatment. The subgroup analysis of geographic regions and GC subtypes was further reported in the 2012 American Society of Clinical Oncology (ASCO) gastrointestinal cancers symposium. For both Asian and non-Asian patients, most patients have type 2 GC (52.1 %) or type 3 (38.3 %) GC, and those with type 2 GC had worse outcome than type 3 GC (median OS 10.3 vs 11.7 months; HR = 0.82; 95 % CI 0.68–1.00). For non-Asian patients, prognosis is worse than Asian patients (8.0 vs 11.1 months; HR = 0.68; 95 % CI 0.53–0.89), and those with GC type 2 (6.5 vs 9.9 months; HR = 0.68; 95 % CI 0.48–0.97) and type 3 (9.0 vs 11.7 months; HR = 0.72; 95 % CI 0.48–1.07) appear to benefit from bevacizumab, making bevacizumab more promising in non-Asian patients [20].

### Ramucirumab

Ramucirumab is a fully humanzed mAb targeting VEGFR2 [21]. Several trials are onging, including a randomized phase II clinical trial of mFOLFOX6 (oxaliplatin, 5-FU, folic acid) chemotherapy plus Ramucirumab versus mFOLFOX6 plus placebo for advanced gastro-esophageal adenocarcinoma, a randomized phase III study of paclitaxel with or without Ramucirumab in patients with metastatic gastric adenocarcinoma after failure of first-line therapy with platinum and fluoropyrimidine, and another phase III study of Ramucirumab eing compared with best supportive care (BSC) in patients with gastric cancer and adenocarcinoma.

### Apatinib

Apatinib is a relatively "clean" TKI that selectively targets VEGFR-2 [22]. A randomized, three-arm phase II trial of apatinib as a third-line treatment for patients with advanced GC included 141 patients who received apatinib (850 mg. qd.), apatinib (425 mg. bid.), or a placebo. The study was reported during the 2011 ASCO Annual Meeting, as follows: ORR of 0 (placebo), 6.38 (apatinib 850 mg qd), and 13.04 % (apatinib 425 mg bid), respectively [23]. The disease control rate was 10.42, 42.55, and 39.13 %, respectively. The median PFS was 1.4 , 3.4, and 3.4 months, respectively; the median OS was 2.5, 4.8, and 4.3 months, respectively. Primary adverse reactions included hypertension and hand-foot syndrome. A phase III clinical trial comparing apatinib to placebo in a 3^rd^-line setting in advanced GC is currently being conducted in China. The enrollment target is 270. The patients are randomized to apatinib 850 mg qd p.o. or placebo and are treaed until disease progression, intolerable toxicity or withdrawal of consent. The trial was estimated to be finished in June 2012.

### Sorafenib

Sorafenib, originally developed for the treatment of kidney cancer, is a multi-target TKI, targeting VEGFR, platelet-derived growth factor receptor (PDGFR), and RAF pathways. It possesses two antineoplastic pathways: one acts directly through the inhibition of tumor proliferation by blocking the RAF/MEK/ERK-mediated cell signaling pathway, and the other indirectly inhibits angiogenesis by blocking VEGFR and PDGFR [24]. Based on the data derived from hepatocellular carcinoma trials, several studies were designed to investigate the role of sorafenib in advanced GC. The results of a phase II study of sorafenib in combination with docetaxel and cisplatin as a treatment for chemo-naive metastatic or local advanced unresectable GC or GEJC patients has been reported [25]. The study enrolled 44 patients who received sorafenib 400 mg bid, in combination with docetaxel 75 mg/m^2^ d1 and cisplatin 75 mg/m^2^ on day 1 in a 21-day cycle. The ORR was 41 %, the median PFS was 5.8 months, and the median OS was 13.6 months. The authors concluded that sorafenib, combined with docetaxel and cisplatin, was effective and tolerable as a treatment for GC or GEJC . However, this study’s results did not indicate superiority over historical data from the docetaxel and cisplatin combination chemotherapy, thereby prompting no further clinical development of sorafenib in GC.

### Sunitinib

Sunitinib blocks angiogenesis by inhibiting targets such as VEGFR, PDGFR, RET and KIT. It is similar to sorafenib in activity, as reported in different tumor types than those observed in sorafenib treatment in both pre-clinical and clinical settings [26]. In a phase II trial, sunitinib monotherapy was conducted on 52 patients with chemo-refractory advanced GC, resulting in a median OS of 5.8 months, displaying less effectiveness than anticipated [27]. A single-arm, monotherapy phase II study was performed in 78 patients as 2^nd^-line treatment. Two patients (2.6 %) had partial responses and 25 patients (32.1 %) had a best response of stable disease for ≥6 weeks. The median PFS was 2.3 months and median OS was 6.8 months. This study showed little clinical value in monotherapy setting [28]. At this time, there is no plan to move forward with sunitinib in further clinical investigations of GC.

### Cediranib

Cediranib, also known as AZD2171, is a highly potent inhibitor of VEGFR-1 and VEGFR-2, which displays activity against c-Kit and PDGFR-β [29]. A phase I study of cediranib in combination with cisplatin plus fluoropyrimidine (S-1 or capecitabine) was performed on 14 Japanese patients with treatment-naive advanced GC [30]. This study demonstrated good tolerability with the most common adverse side effects being decreased appetite, fatigue and nausea. Furthermore, preliminary efficacy evaluation showed one confirmed and three unconfirmed partial responses that were ongoing at the time of data cut-off. Further investigation is expected to continue in the future.

### EGFR-targeting agents

Epidermal growth factor receptor (EGFR) exists on the cell surface and is activated by the binding of specific ligands, including epidermal growth factor (EGF) and transforming growth factor alpha. EGFR possesses an intracellular tyrosine kinase domain that, upon activation, may initiate downstream signaling, ultimately resulting in DNA synthesis and cell proliferation. Inhibition of EGFR activation disturbs downstream signaling and prevents tumorigenesis [31]. The prognostic role of EGFR remains controversial, and further investigation is needed [32-37]. Anti-EGFR mAbs and TKIs are currently undergoing clinical trials for GC patients.

### Cetuximab

Cetuximab is a chimeric (mouse/human) mAb targeting to EGFR. Monotherapy with cetuximab for treatment of advanced GC showed minimal activity [38]. Trials investigating cetuximab treatment after first-line chemotherapies were reported with moderate activities [39,40]. In first-line settings (Table 2), in several phase II trials, cetuximab has shown some encouraging results when combined with various chemotherapeutic agents. Discrepancies among these results may be due to the differences in cytotoxic compounds. The phase III trial (EXPAND) will assess the efficacy of cetuximab in combination with cisplatin and capecitabine as a first-line treatment for patients with advanced GC or GEJC. Results are expected to be released later this year.

**Table 2:** Phase II, first-line clinical trials of cetuximab in combination with various chemotherapeutic agents

**Agents**	***n*(tumor site)**	**RR (%)**	**PFS/TTP (month)**	**OS (month)**
mFOLFOX6 + cetuximab[41]	50 (GC)	50	5.5	9.9
Cisplatin + docetaxel + cetuximab[42]	72 (GC or GEJC)	41.2	5	9
Cetuximab + FOLFOX4[43]	25 (GC)	36	6.5	10.6
Oxaliplatin + irinotecan + cetuximab[44]	51 (GC)	63	5.8	8.9
Oxaliplatin + folic acid + 5-FU + cetuximab[45]	52 (GC)	65	7.6	9.5
Oxaliplatin + capecitabine + cetuximab[46]	44 (GC)	52.3	6.5	11.8
Irinotican + folic acid + 5-FU + cetuximab[47]	49 (GC or GEJC)	46	9	16.5
Epirubicin + cisplatin +5-FU + cetuximab FOLFOX + cetuximab Irinotican + cisplatin + cetuximab[48]	245 (GC or GEJC) randomized	58 51 38	5.6 5.75	10 10 8.6
Cisplatin + 5-FU + LV + cetuximab[49]	35 (GC)	68.6	11	14.5
Oxaliplatin + irinotecan + cetuximab[50]	51 (GC)	63	5.8	8.9
Docetaxel + oxaliplatin Docetaxel + oxaliplatin + cetuximab[51]	150 (GC or GEJC) randomized	24 29	4.7 5.1	9 8.5

### Panitumumab

Panitumumab is a fully humanized IgG2 mAb targeting EGFR. Available data for panitumumab in GC is limited. Based on data from metastatic colorectal cancer [52-54], panitumumab was investigated in a dose-finding trial in gastric cancer to determine the optimal dosage when combined with EOX regimen: epirubicin, oxaliplatin, and capecitabine. Results indicated that a dose of 9 mg/kg of panitumumab every 3 weeks, when combined with EOX, meets the acceptable safety profile; it is currently being used in an ongoing phase II/III REAL-3 trial, which is a randomized, open-label trial of the efficacy of EOX with or without Panitumumab as first line therapy for advanced esophagogastric cancer [55].

### HER2-targeting agent

HER2 is a cell membrane surface-bound receptor tyrosine kinase that is normally involved in the signal transduction pathway leading to cell growth and differentiation. The reported expression of HER2 in GC patients and its correlation with prognosis has varied across different studies [34,56-58]. However, pre-clinical trials have shown the efficacy of suppression of tumor growth in human GC over-expressing HER2 by anti-HER2 antibody, making HER2 a potential target [59,60]. Clinical investigation in GC with HER2 targeting by either mAb or TKI has been actively pursued.

### Trastuzumab

Trastuzumab is a HER2 mAb that induces antibody-dependent cellular cytotoxicity, inhibits HER2-mediated signaling, and reduces the number of cell surface receptors by increasing endocytosis [61]. A multi-center, randomized, controlled phase III clinical study (ToGA) was reported in 2010, with a total of 3807 advanced GC or GEJC patients enrolled. Of these patients, 810 (21.2 %) were positive for HER2 expression (FISH-amplified or IHC 3+ and above). Among the 810 patients, 594 were randomly assigned to receive chemotherapy treatment (cisplatin plus 5-FU or capecitabine administered every 3 weeks for a maximum of 6 cycles) or chemotherapy plus trastuzumab treatment (administered at a dose of 8 mg/kg on day 1 of the first cycle, followed by 6 mg/kg every 3 weeks until the event of disease progression, unacceptable toxicity or consent withdrawal). Median OS was 13.8 months in patients assigned to trastuzumab plus chemotherapy, compared with 11.1 months in those assigned to chemotherapy alone (HR 0.74; 95 % CI 0.60 - 0.91; p = 0.0046). The efficacy result translated into a 26 % reduction in the death rate. Adverse effects were comparable between the two treatment groups [4]. Based on the findings of this trial, trastuzumab, in combination with chemotherapy has been approved by US FDA and European Medicines Agency as first-line therapy in HER2 positive GC and GEJC. Notably, HER2-positivity rates were found to be significantly higher in GEJC than GC (33.2 % vs 20.9 %; p < 0.001), and in intestinal than diffuse/mixed cancer (32.2 % vs 6.1 %/20.4 %; p < 0.001) [62].

### Lapatinib

Lapatinib is an oral TKI which inhibits both EGFR and HER2 kinases. The phase II trial of lapatinib monotherapy as first-line therapy in 47 patients with advanced GC demonstrated excellent tolerability and moderate activity (median time to treatment failure was 1.9 months and OS was 4.8 months). Analyzing potential biomarkers revealed that patients with higher HER2 or lower interleukin-8 gene expression levels experienced prolonged OS [63]. In a phase II trial of capecitabine and lapatinib combination as first-line treatment in 58 patients with GC (76 %) or GEJC (24 %), 24 % of patients showed partial responses (17 % confirmed, 7 % unconfirmed), 36 % showed disease stabilization and 26 % showed disease progression [64]. The LOGiC study (Lapatinib Optimization Study in HER2 Positive Gastric Cancer), a phase III global study, designed to evaluate clinical endpoints and safety of chemotherapy plus lapatinib, has reached its accrual goal and is ongoing for follow up. Efficacy data is anticipated to be released in late 2012. An ongoing TYTAN trial is a randomized, open-label, phase III study of Lapatinib in combination with Paclitaxel versus Paclitaxel alone in the second line setting of erbB2 amplified advanced GC.

### Other targeted agents

#### Everolimus

The mTOR is involved in several important intracellular signal transduction pathways that regulate cell survival, hyperplasia, apoptosis, and other important physiological functions critical to tumorigenesis and cancer development, thus providing as a potential anti-cancer therapy [65]. The first mTOR targeting agent was everolimus, an oral mTOR serine/threonine kinase inhibitor, whose anti-GC activity was observed in both pre-clinical and phase I studies [65-67]. In a phase II study of everolimus in 53 patients with previously treated metastatic gastric cancer, the results showed that the DCR was 56.0 % (95 % CI, 41.3 % to 70.0 %) and median PFS was 2.7 months (95 % CI, 1.6 to 3.0 months). At a median follow-up of 9.6 months, the median OS was 10.1 months (95 % CI, 6.5 to 12.1 months) and good tolerability was noted [68]. Based on the promising results from several phase II trials, an international phase III trial was launched in June 2009. A total of 656 patients with advanced GC previously treated with 1 or 2 lines of systemic chemotherapy from 23 countries were randomly treated with everolimus or BSC plus a placebo. Data released from the 2012 ASCO Gastrointestinal Cancers Symposium indicated that the median OS was 5.4 months with everolimus, vs. 4.3 months with placebo treatment (HR, 0.90; 95 % CI, 0.75-1.08; P = 0.1244) [69]. Median PFS per local investigator assessment was 1.7 months with everolimus vs. 1.4 months with placebo (HR, 0.66; 95 % CI, 0.56-0.78; p < 0.0001). PFS was improved significantly by everolimus, although the OS was not.

#### Onartuzumab

c-Met is an oncogene encoding a membrane tyrosine kinase receptor – hepatocyte growth factor receptor (HGFR). HGFR plays an important role in tumor development through activation of key oncogenic pathways, angiogenesis, and tumor metastasis [70]. In gastric cancer patients, amplification and expression of c-Met often indicates poor prognosis [71-74]. Several types of targeted agents have been developed for c-Met pathway. Onartuzumab is a humanized monoclonal antibody directed against HGFR. A phase II clinical trial of Onartuzumab in combination with mFOLFOX6 in patients with metastatic HER2-negative gastro-esophageal cancer is to be launched soon.

#### Vorinostat

Vorinostat is a HDAC inhibitor, also known as suberoylanilide hydroxamic acid. Pre-clinical studies have shown that vorinostat has potential anti-GC activity, due to a synergistic effect exhibited when combined with taxane [75,76]. A phase I/II study of vorinostat plus capecitabine and cisplatin is currently recruiting patients for first line treatment of metastatic or recurrent GC.

#### Future Perspectives

Contemporary targeted cancer therapy has progressed rapidly over the past decade. Unfortunately, GC remains a major challenge with fewer encouraging targeted treatments than other cancers have. The addition of trastuzumab to combination chemotherapy is now considered a standard first-line treatment for HER2 positive advanced GC patients, and this treatment is still under investigation for potential use in perioperative settings [4]. Lapatinib is currently the most promising new agent for treatment of gastric cancer, in the light of the ToGA trial. Agents targeting human EGFR remain very controversial in treating gastric cancer. Cetuximab has exhibited the potential for GC treatment, which functions by binding to the ubiquitous HER1(EGFR); based on success in treating colorectal cancer patients, cetuximab is currently one of the promising new candidates for treatment of gastric cancer due to its very low mutation rate of K-RAS.

Anti-angiogenic therapy has achieved successful results in several cancer types, including colorectal, hepatocellular, and kidney cancer; however, despite tremendous effort in the past decade, many trials demonstrated only marginal effectiveness over existing treatments. An AVAGAST trial recently released results, indicating negative OS data [19] similar to study results from sorafenib and sunitinib. The primary reason for the disappointing results of anti-angiogenesis treatment is the lack of predictive biomarkers for the investigated drugs. Although biomarkers such as serum VEGFA and microvessel density have repeatedly been cited as potentially useful predictive markers for the effectiveness of anti-angiogenic therapy, they remain unconfirmed by any phase III trial. This makes it difficult to determine which agents are potentially most beneficial to certain patients. Based on the results of the investigations in lung cancer and breast cancer, further clinical analysis of biomarkers may lead to a better understanding of GC outcomes and appropriate treatment selection. Not limited to anti-angiogenic therapy, “Personalizing cancer care” is the goal for all targeting agents, in order to achieve both improved and optimal results based on effective biomarker selection [77].

The majority of existing targeting agents focus on both EGF/VEGF and their receptors, but contemporary research has revealed many new pathways related to tumor angiogenesis and proliferation, providing numerous new potential targets. Trials are already underway to test potential targeting agents focusing on the downstream components of VEGFR/EGFR, such as inhibitors of mTOR, c-Met, and HDAC. The phase III trial of everolimus has reported prolonged PFS at ASCO Gastrointestinal Cancers Symposium this year.

Drug resistance is a critical issue in the development of targeted agents. Effective treatment may involve a combination of different targeted agents, chemotherapies, or it may require the use of target agents as part of a complex approach to cancer treatment utilizing multiple modalities.

## Abbreviations

GC, Gastric cancer; 5-FU, 5-fluorouracil; HER2, Human epidermal growth factor receptor 2; VEGF, ,Vascular endothelial growth factor; mTOR, Mammalian target of rapamycin; HDAC, Histone deacetylase; VEGFR, Receptor of vascular endothelia growth factor; MAb, Monoclonal antibody; TKI, Tyrosine kinase inhibitor; GEJC, Gastro-esophageal junction cancer; ASCO, American Society of Clinical Oncology; OS, Overall survival; PFS, Progression-free survival; ORR, Overall response rate; DCR, Disease control rate; PDGFR, Platelet derived growth factor receptor; EGFR, Epidermal growth factor receptor; EGF, Epidermal growth factor; BSC, Best supportive care.

## Competing interests

The authors declare that they have no competing interests.

## Authors’ contributions

All authors participated in the drafting and editing of the manuscript. All authors read and approved the final manuscript.
